# Angiographic Evidence of a Purely Pial Bihemispheric Intracranial Hemangiopericytoma

**DOI:** 10.1155/2016/5245078

**Published:** 2016-01-05

**Authors:** Nathaniel Stetson, Sudhakar Vadivelu, Jiang Y. Li, Avi Setton, David J. Chalif

**Affiliations:** ^1^Cushing Neuroscience Institute and Department of Neurosurgery, Hofstra North Shore-LIJ School of Medicine, Manhasset, NY 11030, USA; ^2^Cincinnati Children's Hospital Medical Center, University of Cincinnati College of Medicine, 3333 Burnet Avenue, MLC 2016, Cincinnati, OH 45229-3039, USA

## Abstract

*Background*. Classification of hemangiopericytoma (HPC) has evolved to a mesenchymal, nonmeningothelial grade two or three neoplasm according to the World Health Organization; however its blood supply has always been defined by dual origin, pial and dural contribution.* Case Description*. We present the case of a patient with an intracranial HPC with only pial vascular supply. Angiography confirmed the lack of dural supply to this bihemispheric intracranial mass. Subsequent histologic examination confirmed the diagnosis of hemangiopericytoma. Angiographic evidence here is atypical of the natural history of hemangiopericytomas with dual vascular supply and was critical in the decision-making towards surgical resection without tumor embolization.* Conclusion*. Data presented suggests the lack of dural vascular supply alone does not rule out the diagnosis of hemangiopericytoma.

## 1. Introduction

In 1942, Stout and Murray [1] first termed “hemangiopericytoma” as tumors that presented with an appearance of soft tissues anywhere on the body, occurring wherever capillaries exist [2]. Intracranial hemangiopericytomas (meningeal) are generally considered a more aggressive phenotype in comparison to the solitary fibrous phenotype based on observations of increased cellularity and reduced collagen bands [3]. The natural history accounts for an incidence of 2-3% of all primary meningeal tumors [4], and they are believed to arise from Zimmerman pericytes, small muscular cells lining capillary and postcapillary venule walls [1]. HPCs are aggressive tumors with high rates of recurrence and frequent extracranial metastasis.

Originally thought to be a variant of meningioma, HPC only recently has been delineated as its own histological entity and is now classified as mesenchymal and nonmeningothelial [5]. HPC is graded as a World Health Organization grade II neoplasm, with an anaplastic variant as grade III [6, 7]. Based on CT and MRI evidence, the differential diagnosis includes meningioma, solitary fibrous tumor, lymphoma, sarcoidosis, and gliosarcoma [8]. However, angiographic evidence of HPC demonstrates dual blood supply from internal carotid or vertebral arteries (pial) and external carotid arteries (meningeal-dural), with dominant supply from the internal carotid circulation [2, 8].

## 2. Case Study

**Figure 1:**
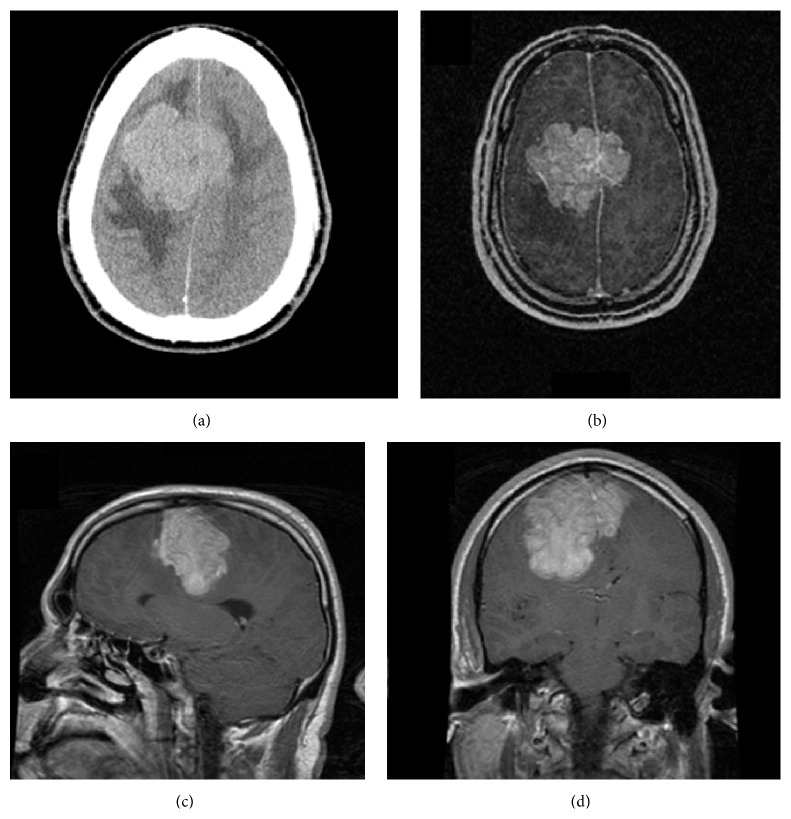
Axial, noncontrast CT scan (a) demonstrating a hyperdense, bifrontal, parasagittal lesion with surrounding vasogenic edema. T1 weighted, postcontrast MRI images showing (b) axial, (c) sagittal, and (d) coronal views of the mass.

A 35-year-old right handed male presented with two months of progressively worsening headaches and otherwise unremarkable history. Neurological examination revealed no focal deficits. His evaluation included a negative metastatic workup, a CT scan showing a hyperdense, bifrontal, parasagittal lesion with surrounding vasogenic edema causing significant mass effect (Figure 1(a)) without evidence of bony erosion. A MRI redemonstrated the bilateral tumor with the mass as isointense to grey matter on T1 weighted images and mildly hyperintense on T2 weighted images and did not appear to have any obvious dural attachment. An obvious arachnoid plane completely encased the mass within the brain parenchyma. Postcontrast T1 weighted images demonstrated avid enhancement, a lobulated border, and numerous flow voids (Figures 1(b)–1(d)).

**Figure 2:**
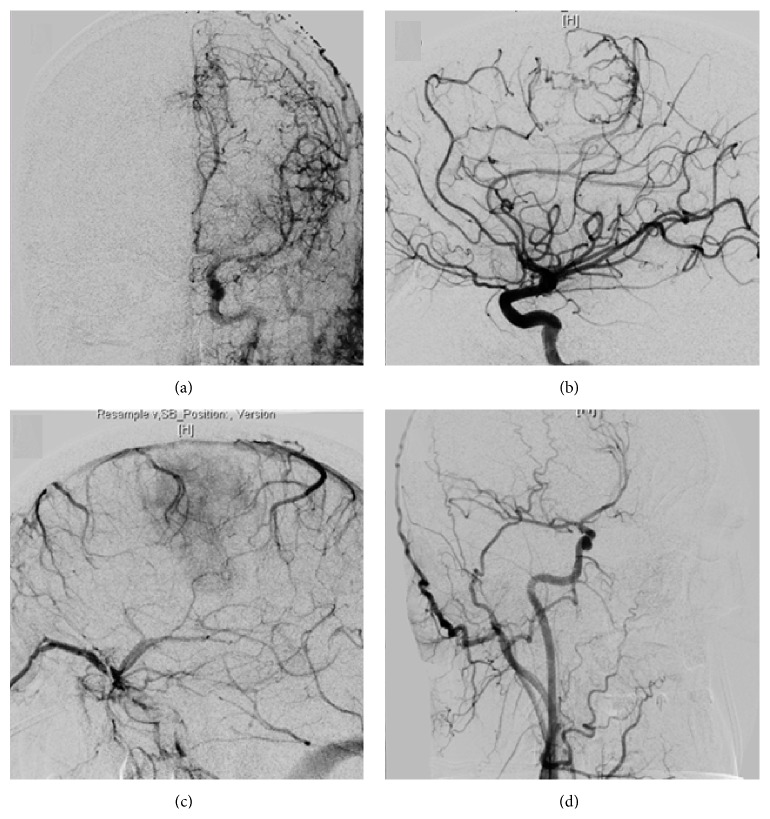
Cerebral angiogram in (a) AP and (b) lateral common carotid injections demonstrating blood supply of the tumor crossing midline from the left distal ACA. The left carotid venous phase (c) shows a dense tumor blush. An oblique right common carotid injection (d) demonstrates no contribution to the tumor.

Cerebral angiography demonstrated only pial blood supply. The arterial and capillary phase from the left internal carotid angiogram demonstrated a number of small “corkscrew” vessels originating from the distal left callosomarginal artery, with no obvious contribution from either anterior falcine artery, as expected from a parasagittal-presumably dural based lesion. The right carotid injection demonstrated a deviated distal ACA territory but no pial supply to the tumor blush (Figure 2). Selective injection of both ECAs demonstrated normal appearing frontal and parietal meningeal branches without dural supply to the posterior frontal mass. Angiographic anatomy demonstrated a purely intra-axial mass. Due to the limited supply from small-caliber pial vessels, we did not pursue preoperative embolization.

The patient underwent a gross total resection of the mass. At surgery, there was no evidence of hypertrophy of any meningeal vessels on both the inner and outer surfaces of the dura which appeared completely normal in color and character. The tumor was subpial and presented as a dark reddish-purplish hypervascular mass. The tumor clearly invaded the inferior aspect of the falx with extension under the free edge toward the contralateral side. After resection of the bilateral tumor, the inferior half of the falx in the region of the mass was resected in totality. Postoperatively, the patient woke up without neurological deficit and progressed with a benign course throughout his inpatient hospital stay. He was discharged home on postoperative day four.

Gross examination of the tumor specimen showed a fleshy, multilobulated tumor that was distinguishable from normal brain but almost entirely encapsulated within the parenchyma. The tumor appeared to be growing around the falx cerebri with one area of invasion.

**Figure 3:**
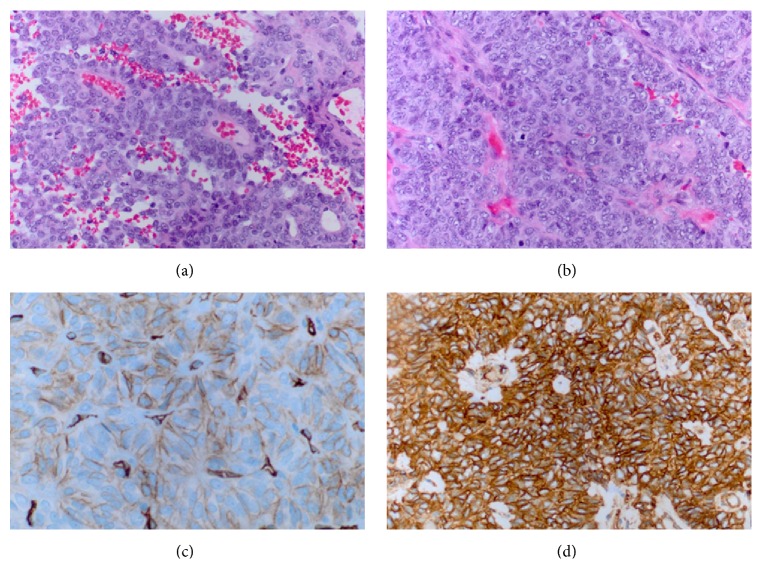
Photomicrograph of tissue sections showing papillary and clear cell morphology with hypervascularity, but no characteristic staghorn appearance (a). Necrosis, intratumoral hemorrhage, nuclear atypia, and frequent mitotic figures were seen (b) and there was patchy and strong CD34 staining (c), as well as strong and diffuse CD99 staining (d).

Histological examination revealed a malignant neoplasm with marked hypercellularity and nuclear atypia, hypervascularity, necrosis, and intratumoral hemorrhage consistent with a WHO 2007 grade III anaplastic hemangiopericytoma [7]. Mitotic figures were easily seen (more than 5 mitoses/10 HPF). Clear cell morphology and papillary formation were noted along with dilated thin vasculature, but no characteristic staghorn pattern. The tumor focally attached to the surface of brain. Immunostains were performed and showed that tumor cells were positive for CD99 (strong and diffuse), CD34 (patchy and strong), and vimentin and reticulin (Figure 3) but negative for EMA, PR, S100, HMB-45, Melan A, pancytokeratin AE1/AE3, and Cam5.2. The Ki-67 labeling index was markedly elevated (up to 10–15% in the highest areas).

## 3. Discussion

Hemangiopericytomas present rarely as intracranial tumors and little is known regarding their pathogenesis and development. They are usually low grade malignant tumors that have a tendency to continue growing, even after gross total resection, and can metastasize to numerous extracranial locations [9, 10]. A growing number of reports suggest a shift from meningeal origin to a nonmeningeal origin [1]. Angiographically, some small series of HPCs have found either (1) a primary dural/external carotid blood supply or (2) a dual internal and external carotid blood supply, with the predominant contribution being pial [2, 5, 8]. In contrast, Akiyama et al. reported on a series of seven cases and suggested one parasagittal HPC in a 33-year-old male to have only pial blood supply [5]. We document the second HPC case with isolated pial vascular supply. The data presented suggests that the lack of dural supply should not exclude the diagnosis of HPC. We provide further evidence to support an additional nonmeningeal origin in the natural history of HPC.

## 4. Conclusion

This is the second case of a purely pial based HPC. Knowledge of the anatomical variability of the blood supply is essential to the successful endovascular and surgical treatment of these tumors. Preoperative angiography is mandatory. Evidence presented here further suggests that a lack of dural vascular supply does not exclude HPC diagnosis.
